# Non-Sensory Perception and Sensory Appeal of *Zamnè*, *PseudoZamnè*, Traditionally Cooked *Senegalia erythrocalyx* Seeds, and Tempeh According to Burkinabe Consumers

**DOI:** 10.3390/foods12234268

**Published:** 2023-11-26

**Authors:** Moustapha Soungalo Drabo, Korotimi Traoré, Charles Parkouda, Fatoumata Hama-Ba, Aly Savadogo, Katleen Raes

**Affiliations:** 1 Research Unit VEG-i-TEC, Faculty of Bioscience Engineering, Ghent University, Sint-Martens-Latemlaan 2B, 8500 Kortrijk, Belgium; moustaphasoungalo.drabo@ugent.be; 2 Laboratory of Applied Biochemistry and Immunology, Department of Biochemistry and Microbiology, University Joseph Ki-Zerbo, Ouagadougou 03 BP 7021, Burkina Faso; alysavadogo@gmail.com; 3 Department of Food Technologies, Institute of Research in Applied Sciences and Technologies, Ouagadougou 03 BP 7047, Burkina Faso; timitraor@gmail.com (K.T.); cparkouda@gmail.com (C.P.); hamafatou@yahoo.fr (F.H.-B.)

**Keywords:** consumer perception, indigenous food, *Kumatiya*, *Senegalia* seeds, sensory properties, tempeh, *Zamnè*

## Abstract

The lack of adequate knowledge of the culinary and sensory properties of most indigenous and wild foods hampers their promotion in human diets and the market. In the present study, 80 Burkinabe volunteers evaluated the sensory appeal and attributes of three selected *Senegalia* seed species (*Zamnè*, *pseudoZamnè* or *Kumatiya*, and *S. erythrocalyx*) and their food formulae (traditionally cooked, harvested as green and fresh legumes, and fermented as tempeh) using the nine-point hedonic scale and check-all-that-apply questionnaire. They found that the traditionally cooked *Zamnè*, *pseudoZamnè* or *Kumatiya*, and derived tempeh had good sensory appeal (scoring between 5 and 7) and subtle alkaline and nutty tastes. However, an appreciable number (32%) of the participants were unaccustomed to tempeh and gave very low scores (2.5–3.4) for all the tempeh products. In contrast, the traditionally cooked seeds of *Senegalia erythrocalyx* and the green and fresh *Zamnè* evoked bitter and sour off-tastes, respectively, and were not much appreciated (scoring 4). The present study provides unprecedented insight into consumers’ non-sensory perceptions and the culinary and sensory properties of *Senegalia* seed foods, which will be essential for their valorization, branding, and marketing.

## 1. Introduction

*Senegalia* seeds are wild legumes tapped by indigenous people in the arid and semi-arid tropics as famine foods and traditional foods [1,2,3]. They have prospects as nutraceuticals or health-promoting foods and appear to be potential sources of high-quality protein (10–20 g/100 g dry matter), dietary fibers (20–50 g/100 g dry matter), and bioactive phytochemicals [1,2,4,5,6,7,8]. However, although some seed species have become delicacies (i.e., *Zamnè* and *Kumatiya*) locally, they have remained overlooked and underresearched for a long time. Particularly, the knowledge of their food uses is sparsely disseminated, and reports on their sensory properties are hardly documented. For example, though *Kumatiya* (i.e., *Senegalia senegal* seeds) is considered a delicacy in the state of Rajasthan (Northern India), it is regarded as a *pseudoZamnè* in Burkina Faso. Needless to say, the *pseudoZamnès* are claimed to be of lower quality compared to *Zamnè* (i.e., *Senegalia macrostachya* seeds), despite the limited knowledge of their culinary, sensory, and nutritional properties. In fact, *Senegalia* seeds are traditionally boiled as legumes and rarely nibbled fresh and green during foraging, harvested fresh and green as a condiment for sauce, or milled and used in bakery products (e.g., cakes and bread). As it stands, *Kumatiya* and *Zamnè* have demonstrated comparable cooking and nutritional properties, and both have shown similar hard-to-cook problems (i.e., resistance to conventional cooking). The hard-to-cook problem obliges a harsh traditional cooking process that compromises *Senegalia* seeds’ nutritional properties [5,9,10].

Considering the hard-to-cook problem, we recently demonstrated the fermentability of *Zamnè*, as a benchmark of edible *Senegalia* seeds, into tempeh [5]. Tempeh is originally a traditional Indonesian food, which is currently receiving attention as a healthy, highly nutritious, and multifunctional food product worldwide [11,12]. As expected, compared to traditional cooking, tempeh fermentation improved the nutritional properties and the digestibility of *Zamnè*. Moreover, since raw *Senegalia* seeds seem edible [2,13], they could also be harvested early as fresh and green vegetables to bypass the hard-to-cook problem. Suffice it to say, *Senegalia* seeds are potential food resources that can support food diversification and provide a safety net in the arid and semi-arid tropics. On the other hand, tempeh fermentation is a promising processing alternative that could help valorize them. However, as noted, there is sparse information on the culinary properties, sensory properties, and non-sensory perceptions (such as a priori and historical imprints) of *Senegalia* seed foods, and tempeh is still an unaccustomed food product outside Indonesia [11]. However, while the promotion of *Senegalia* seeds in human diets and markets requires accurate language to describe them, it is essential to select product formulations that align with the food culture and the sensory preferences of the targeted consumers. Therefore, the objective of this study was to assess the non-sensory perceptions and the sensory profiles and appeals of selected *Senegalia* seed species and their traditional cooking and processing alternatives (i.e., early harvesting and blanching as green legumes, and tempeh fermentation) for consumers in Burkina Faso. Moreover, the psychographic traits of the participants in the study, such as their a priori and overall attitudes toward the products, were derived.

## 2. Materials and Methods

### 2.1. Ethical Clearance and Recruitment of the Participants

This study was approved on the 5th of January 2022 by the ethics committee for health-related research of the Health Ministry of Burkina Faso (clearance number: CERS 2021-12-290). Then, volunteers for the sensory test were invited from the general population in Ouagadougou, students from University Joseph Ki-Zerbo, and personnel from the Department of Food Technology (Institute of Research in Applied Sciences and Technologies, Burkina Faso) using flyers. The flyers provided brief information on the study, illustrations of the products, and the requirements for participation in the study, i.e., health status (such as no food allergies, smoking, and respiratory infections), good sensory acuity, age > 18 years, and familiarity with *Zamnè*. The volunteers were not given any cash incentive for their participation.

### 2.2. Products

Mature and dry *Zamnè* (*Senegalia macrostachya*) and *pseudoZamnè* (*Senegalia senegal*) seeds (5 kg each) were harvested from the wild (N 13.09 W 03.12, Burkina Faso) in December 2018, and specimens were identified and deposited at the Herbarium INFOBIO (reference N° 6887 and 6886) of University Joseph Ki-Zerbo. Mature and dry *Senegalia erythrocalyx* seeds (1 kg) and soybeans (2 kg) were purchased from the National Centre of Forest Seeds (Centre National de Semence Forestière, CNSF, Burkina Faso) and a local market in Ouagadougou, respectively. All of the samples were vacuum-sealed in airtight plastic bags and stored at −20 °C until analysis. It is important to note that no record of human consumption of *S. erythrocalyx* seeds was found [2]. The species was included considering its food potential, i.e., specific compositional properties and cookability [6,14].

**Figure 1:**
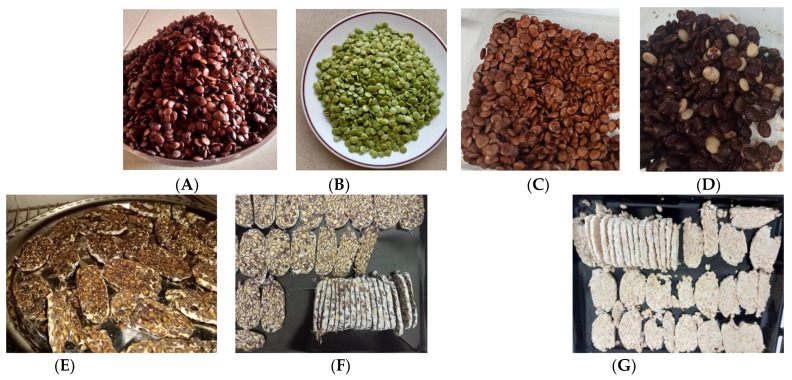
Illustration of the products: (**A**) traditionally cooked *Zamnè* (*Senegalia macrostachya* seeds); (**B**) blanched green *Zamnè*; (**C**) traditionally cooked *pseudoZamnè* or *Kumatiya* (*Senegalia senegal* seeds); (**D**) traditionally cooked *Senegalia erythrocalyx* seeds; (**E**) fresh *Zamnè* tempeh; (**F**) *Zamnè* tempeh crisps; (**G**) fresh soy tempeh (tempeh standard).

One kilogram each of *Zamnè*, *pseudoZamnè*, and *S. erythrocalyx* seeds was cooked the same day (early morning) as the sensory test, following the traditional cooking process of *Zamnè* (i.e., boiling of the seeds in 1% *w*/*v* potash solution) [9]. In addition, 1 kg each of *Zamnè* and soybeans were fermented into fresh tempehs using *Rhizopus oryzae* starter culture (Culture for Health, Morrisville, NC, USA), as also described previously [5]. It was ensured that the fermentation was completed (48 h) on the morning of the testing day. In addition, 1 kg of *Zamnè* tempeh was prepared, sliced, and dried at 60 °C for 24 h to obtain crispy tempeh slices (as a new snack). The tempeh crisps were prepared one day before the test and stored at room temperature. Finally, mature and still green and fresh *Zamnè* (3 kg) was harvested at the end of November 2021 at the urban zoo–botanical park Bangr-Weogo (Ouagadougou, Burkina Faso), washed, boiled (98 ± 2 °C) for 10 min in plain water on the day of the harvest, transferred into glass bottles (with the seed covered with water 2 inches above), and steamed for 15 min. The thus-blanched and -canned green *Zamnè* was stored at 4 °C in order to keep it fresh and green until the day of the test (i.e., one month later). It was then drained on the day of the test before being served to the participants. The products are illustrated in Figure 1.

### 2.3. Assessment of the Products

#### 2.3.1. Accommodation of the Participants and Pre-Questionnaire

The study was presented to the volunteers as a sensory analysis of *Zamnè*, *pseudoZamnès*, and tempeh (i.e., a novel product to them), and they were invited to the Department of Food Technology (Institute of Research in Applied Sciences and Technologies, Burkina Faso). The instructions for the test were given in a meeting room, where the volunteers were received and asked to sign a voluntary consent. The volunteers were asked when they ate their last meal and then immediately guided to the sensory test room (~25 °C, noise-free, and adequately lit (daylight-type illumination)) and given individual booths or kept for some time in the meeting room to standardize their stomach-emptying times (2–3 h) before the test, as described by Meilgaard et al. [15]. While waiting for the food products, the participants were invited to respond to a pre-questionnaire, including their gender, age, recent illness, medications, smoking status, food allergies, frequency of consumption of *Zamnè*, a priori opinions on *Zamnè* (i.e., “Are you a fan of *Zamnè*?”), knowledge of tempeh (i.e., “Did you know about tempeh before this study?”), and open-ended questions to determine why they like or dislike *Zamnè* and what they expect from a novel food like tempeh (introduced to them as an Indonesian fermented food with a mold). After that, the sensory test was conducted in one day (with an approximate testing time of 30 min for each participant), and the volunteers were accepted from 9 am until the required number (80) of participants was reached (i.e., 4 pm). The number of participants was determined as described by Gacula and Rutenbeck [16] and Ares et al. [17], in order to detect any minimum difference of 0.6 points on the 9-point hedonic scale and to achieve a reliable configuration of sensory descriptors with the check-all-that-apply questionnaire.

#### 2.3.2. Sensory Test and Overall Attitudes toward the Products

The products were kept at room temperature, served (3 full tablespoons of each cooked/blanched seed product and one slice for each tempeh product) in transparent and disposable plastic cups, labeled with three random digits, and presented to the participants in a monadic series and balanced orders by using the generalized Latin square [18]. The participants were asked to score the appearance, smell, taste, aftertaste, texture (i.e., mouthfeel), and overall sensory appeal of the products using a labeled 9-point hedonic scale (1 = “dislike extremely”, 2 = “dislike very much, 3 = “dislike moderately”, 4 = “dislike slightly”, 5 = “neutral”, 6 = “like slightly”, 7 = “like moderately”, 8 = “like very much”, and 9 = “like extremely”) and then to check all the sensory attributes that apply from a list of 20 sensory descriptors. The sensory descriptors list was constituted after an unstructured survey (including 3 non-timber forest product business holders and 9 individual respondents) to learn how consumers described *Zamnè*, along with preliminary sensory tests of the products by 5 laboratory personnel. The descriptors included 20 general terms on taste, appearance, and texture (i.e., mouthfeel) characteristics. As suggested by Lim, Wood, and Green [19], 3 extra points, though not used by any of the participants, were added to both tails (with the label “most pleasant sensation imaginable” for a score of 12 and “most unpleasant sensation imaginable” for a score of −2) of the scale to reduce the ceiling effect. Additionally, the participants were given mineral water to cleanse their palates between the evaluations of the different products. After the sensory evaluations, the participants were asked to give their overall perceptions (i.e., willingness to buy and willingness to eat as a last resort) of the products using a labeled 5-point scale (1 = “certainly will not buy/eat even if last resort”, 2 = “probably will not buy/eat even if last resort”, 3 = “undecided”, 4 = “probably will buy/eat if last resort”, and 5 = “certainly will buy/eat if last resort”) [20]. Last but not least, space was given to the participants to provide any additional remarks.

### 2.4. Data Collection and Analysis

The data were collected using paper questionnaires, entered into Excel sheets, and analyzed using R version 4.2.0. The sensory appeal scores were subjected to a two-factor (i.e., products and clusters) analysis of variance (ANOVA) and Tukey’s range test. The interrelationships between the scores were subsequently assessed by running Pearson’s correlation test. The clustering of the participants, based on their scoring of the sensory appeal of the products, was assessed by performing k-means clustering, and the number of clusters was determined based on their substantiality (i.e., the size of the clusters) and differentiability (i.e., the conceptual distinction of the clusters) [21]. Afterwards, the frequencies of use of the sensory descriptors at the whole-panel level to describe the products were compared using Cochran’s Q test and Dunn’s test (with Bonferroni-corrected *p*-values). Then, the differences in the frequencies of use of the sensory descriptors between the different panel clusters were determined using Fisher’s exact test. The configurations of the products and the sensory descriptors between the clusters were compared by calculating the RV coefficients. Subsequently, a correspondence analysis (CA) was used to map the relationships between the products and the sensory attributes. Finally, the mean drops in the overall appeal scores (or penalties) due to the deviations in the sensory attributes of the unfamiliar products from the reference product (i.e., *Zamnè*) were calculated as described by Plaehn [22]. Based on Pareto’s principle, the 20% consensus cutoff was applied to determine the relevance of the sensory descriptors and the significance of the penalties [22,23].

## 3. Results

### 3.1. Panel Composition and a Priori Perceptions of the Participants

Table 1 summarizes the panel’s composition. In total, 80 volunteers participated in the study, comprising 61% females, 39% males, and 95% 19–40-year-old adults. The participants were all familiar with *Zamnè*, and 39% of them asserted that they were fans of *Zamnè*, though most (77%) of them were eating *Zamnè* occasionally (i.e., less than once every month). Actually, a very limited number of the participants (<1%) expressed a cultural attachment to *Zamnè*. In contrast, less than 3% of the participants had heard about tempeh before this study but never tried it. Moreover, three participants did not complete the sensory questionnaire and were not included in the sensory data analysis.

Furthermore, the participants expressed their perceptions of the traditionally cooked *Zamnè* and their expectations from any alternative processing of it as follows (Table 2): The fans of the traditionally cooked *Zamnè* (26% of the panel) felt somehow culturally attached to *Zamnè* and perceived it as tasty, wholesome, and nutritionally rich. Very few of them (6%) were aware of the health claims or medicinal properties of *Zamnè*. In contrast, the participants who were not a priori fans of the traditionally cooked *Zamnè* perceived it as tasteless and hardly accessible. Only a few (16%) who were not fans of it noted it as bitter and smelly. Still, the participants expected that any new processing of *Zamnè* should provide better nutritional properties, health benefits, and preparation convenience.

### 3.2. Sensory Appeal of the Products

At the whole-panel level, the traditionally cooked *Zamnè* and the *pseudoZamnè* demonstrated an appreciable sensory appeal and higher scores (6–7) for all of the sensory attributes compared to the blanched green *Zamnè* (score = 3.8), the traditionally cooked *S. erythrocalyx* seeds (score = 4.0), and all of the tempeh products (score = 5.0) (Table S1). Though the blanched green *Zamnè*, the traditionally cooked *S. erythrocalyx* seeds, and the tempeh products received similar scores for their aromas, appearances, and texture, the latter had relatively better overall sensory appeal and slightly higher scores for the taste and the aftertaste. Accordingly, the participants intended to buy the traditionally cooked *Zamnè* and *pseudoZamnè* and showed some reservations about the tempeh products. They were disgusted with the blanched green *Zamnè* and the traditionally cooked *S. erythrocalyx*, as shown by the hesitation (60–70% of the panel) to eat them even if they were last-resort foods (Table S1).

**Figure 2:**
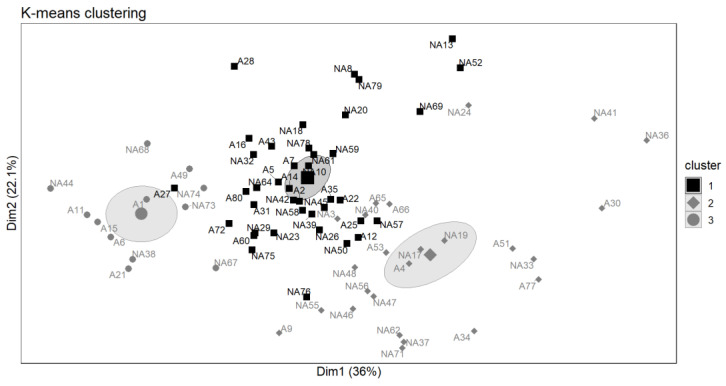
Clustering of the participants based on their scoring of the aroma, appearance, texture, taste, aftertaste, and overall sensory appeal of the products. The participants were encoded as A when they were a priori fans of *Zamnè* and NA when they were not a priori fans of *Zamnè*. Cluster 1 includes 40 participants and counts 39% as *Zamnè* fans. Cluster 2 consists of 25 participants and counts 36% as *Zamnè* fans. Cluster 3 contains 12 participants and counts 45% as *Zamnè* fans.

However, based on the scoring of the sensory attributes and the overall sensory appeal of the products, the participants were segmented into three clusters, and interestingly, the clusters were not associated with their a priori attitudes (i.e., fans or not) to *Zamnè* (Figure 2). In contrast, although 32% of the participants (Cluster 2) were very picky and only liked the traditionally cooked *Zamnè* and *pseudoZamnè*, an appreciable number (52%) of them (Cluster 1) were moderately selective and mainly did not like the blanched green *Zamnè* and the traditionally cooked *S. erythrocalyx* (Table S1). Also, though modest, 16% of the participants found all of the products appealing.

**Figure 3:**
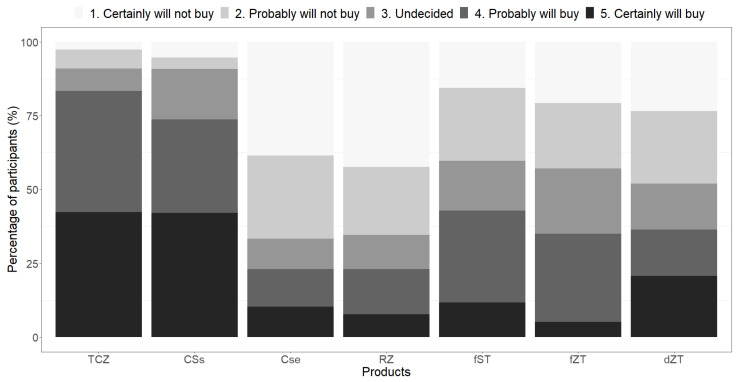

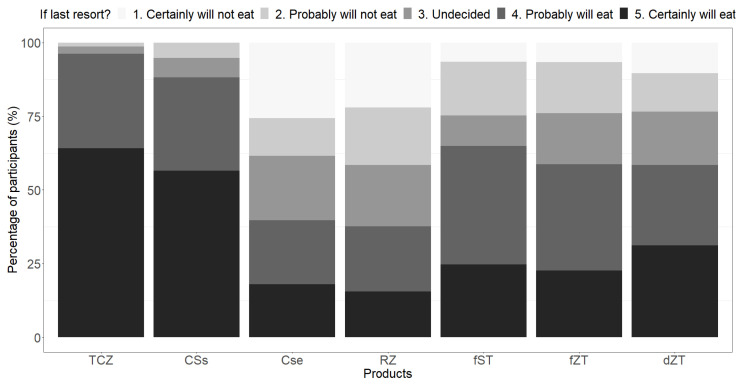
Overall appeal of the products ^Ω^. ^Ω^ Seventy-seven (77) participants scored the products. TCZ, CSs, and CSe refer to the traditionally cooked *Zamnè* (*Senegalia macrostachya* seeds), *pseudoZamnè* (*Senegalia senegal* seeds), and *Senegalia erythrocalyx* seeds, respectively. RZ, fST, fZT, and dZT refer to the blanched green *Zamnè*, fresh soy tempeh, fresh *Zamnè* tempeh, and *Zamnè* tempeh crisps, respectively.

Furthermore, in line with the clustering, the participants showed variable attitudes to the products (Figure 3). While there was unanimity (92% of the participants) in the willingness to buy and eat the traditionally cooked *Zamnè* and *pseudoZamnè*, a lesser (but still appreciable) number of participants (38% and 60%) were willing to buy and eat the tempeh products, respectively. Meanwhile, many participants (77% and 62%, respectively) were not willing to buy the blanched green *Zamnè* and the traditionally cooked *S. erythrocalyx* seeds or eat them even if they were last-resort foods.

### 3.3. Sensory Profile of the Products

**Figure 4:**
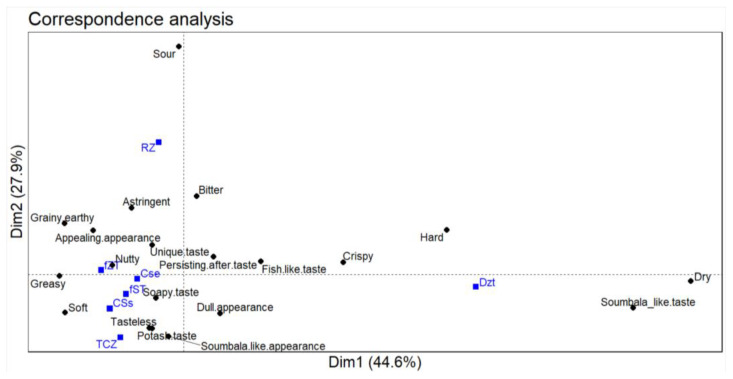
Relationships between the products and their sensory descriptors. TCZ, CSs, and CSe refer to the traditionally cooked *Zamnè* (*Senegalia macrostachya* seeds), *pseudoZamnè* (*Senegalia senegal* seeds), and *Senegalia erythrocalyx* seeds, respectively. RZ, fST, fZT, and dZT refer to the blanched green *Zamnè*, fresh soy tempeh, fresh *Zamnè* tempeh, and *Zamnè* tempeh crisps, respectively. The dimensions (dim) 3, 4, 5, and 6 accounted for 15.7, 8.0, 2.5, and 1.3% of the total inertia, respectively.

The products had quite different sensory profiles, as shown by the significant differences in the frequency of use of all of the sensory descriptors (Table S2) and the correspondence analysis (Figure 4). Up to four major dimensions (with 45, 28, 16, and 8% of the total inertia, respectively) were needed to capture the variances in the frequency of use of the sensory descriptors. Meanwhile, three sensory descriptors (i.e., fish-like taste, astringency, and earthiness) did not receive an appreciable consensus (i.e., less than 20% of the participants) as characteristics of any of the products, considering Pareto’s principle [22]. So, 17 descriptors were variably used to describe the products.

For instance, the traditionally cooked *Zamnè* and *pseudoZamnè* revealed very similar sensory profiles and were asserted to be soft and to have a soumbala appearance and a unique taste with a note of nut and potash or alkali. It is interesting to note that, in contrast to the a priori expectations (Section 3.1), none of the participants noted the bitterness in the traditionally cooked *Zamnè* after the sensory test (Table S2). Moreover, though the traditionally cooked *S. erythrocalyx* seeds were also considered to be soft and with a note of potash, they were identified as greasy, bitter, and with a persistent aftertaste and a dull and (relatively) soumbala appearance. The blanched green *Zamnè*, for instance, was noted as sour, in between soft and hard, but appealing in appearance. Except for the fact that a nutty taste was noted in the fresh *Zamnè* tempeh and not in the fresh soy tempeh, they had relatively similar sensory profiles (particularly a subtle taste and softness). Last but not least, the drying of *Zamnè* tempeh into crisps produced a soumbala-like taste, a persistent aftertaste, and a soumbala and dull appearance.

Furthermore, an appreciable number of the participants (30–60% of the panel) concluded that all of the products had unique tastes. The clusters of the participants differed only in the use of very few descriptors for some of the products (i.e., the potash or alkaline taste for the traditionally cooked *Zamnè* and *S. erythrocalyx* seeds, the lack of taste for the traditionally cooked *pseudoZamnè* and the fresh soy tempeh, and the crispiness and the fish-like taste for the *Zamnè* tempeh crisps) (Table S3). Therefore, an excellent agreement between the clusters and between the participants at the whole-panel level was achieved, as supported by the high RV coefficients (0.93 and 0.79) (*p* < 0.001) between the product configurations and between the descriptor configurations, respectively.

### 3.4. Penalties of the Sensory Attributes on the Overall Sensory Appeal of the Products

**Figure 5:**
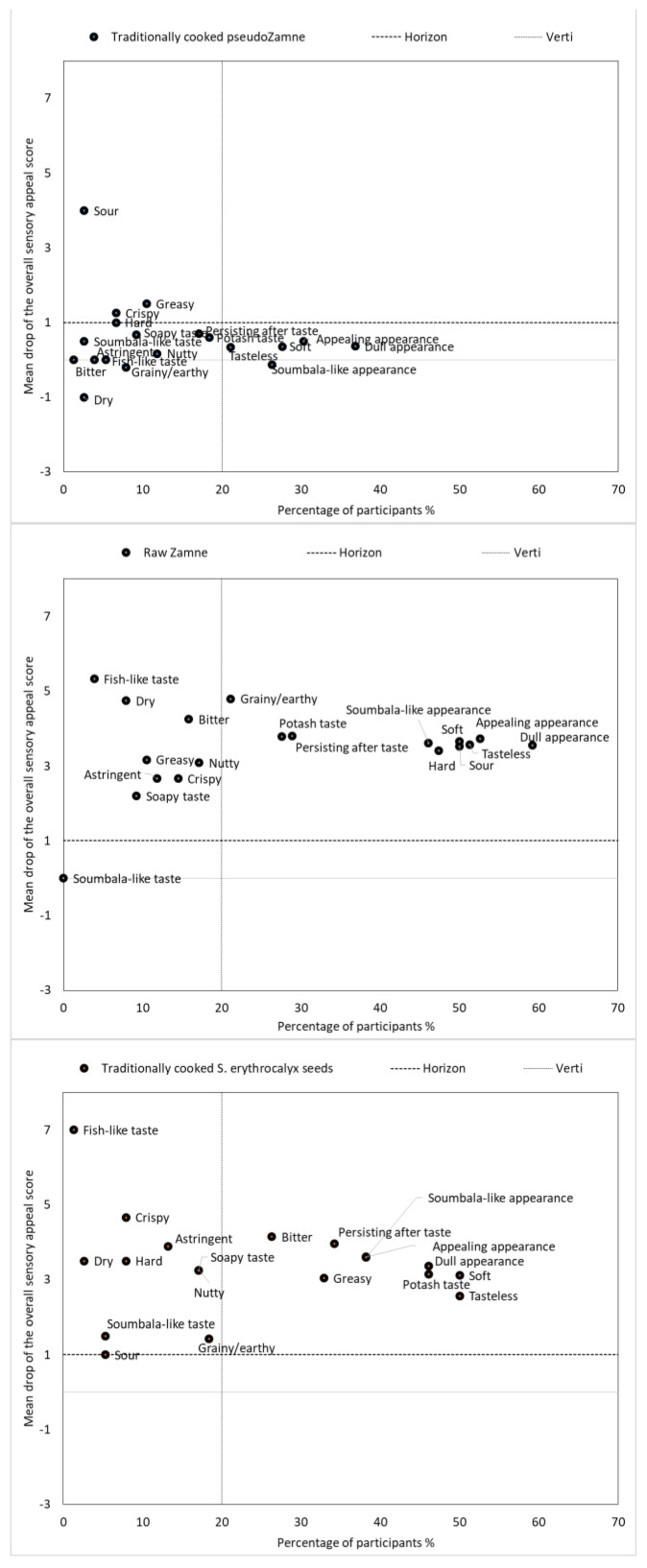
Penalties on the overall sensory appeal of the unfamiliar products as a function of the percentage of participants (N = 77) who checked sensory descriptors differently from the traditionally cooked *Zamnè* (i.e., reference product). Mean drops of more than one point with a consensus of more than 20% of the participants were considered significant (cutoffs indicated by the dashed lines).

Two approaches (i.e., Pearson’s correlation and penalty analysis) were used to assess the influence of the sensory attributes on the overall sensory appeal of the products. As shown by the Pearson’s correlation coefficients, the taste and the aftertaste appeared to be the most determinant factors for the overall sensory appeal and the willingness to buy and eat the products (if as a last resort) (Table S4). They were followed in order by the texture, the aroma, and the appearance of the products. In more detail (Figure 5), in line with Section 3.3, and considering the traditionally cooked *Zamnè* as a reference product, the perception of the hardness and the sourness likely penalized (>3 points) the sensory appeal of the blanched green *Zamnè*. In contrast, the bitterness, the greasiness, the persistence of the aftertaste, and the dull appearance likely penalized (>3 points) the traditionally cooked *S. erythrocalyx* seeds. Since the traditionally cooked *pseudoZamnè* demonstrated a similar sensory profile to the traditionally cooked *Zamnè*, no relevant penalty was determined. Meanwhile, it is important to note that the penalty on the sensory appeal of the tempeh products could not be determined, since the participants were unfamiliar with tempeh and could not define the ideal or reference product, as shown by the similar score (5.0) that they gave to all of the tempeh products, including the (soy) tempeh standard (Table S1).

## 4. Discussion

This study was designed as consumer-based research, including sensory tests (i.e., overall sensory appeal and sensory profile) and psychographic evaluations (i.e., a priori, intention to purchase, and post hoc segmentation). Interestingly, as presented in Section 3.1, the panel was composed as expected (according to an unstructured preliminary survey) and provided a good representation of *Zamnè* consumers (i.e., almost equal numbers of fans and non-fans). The contrast between the number of fans and the frequency (less than once a month) of the consumption of *Zamnè* could be explained by the fact that *Zamnè* is expensive, hard-to-cook, and rarely cooked as a family dish, but mainly being used as a delicacy during social events (where people occasionally get access to it) [24,25]. This observation could be supported by the remarks of the participants on the inaccessibility and the cooking labor of *Zamnè* (Table 2). The low familiarity of the assessors with most of the products is an important limitation of the present study and an inherent concern in the sensory analysis of non-conventional or underutilized food products like *Senegalia* seeds [26]. Furthermore, the observed low cultural attachment, as expressed by the study participants, could be explained by the fact that most (95%) of the participants were of a young generation (<40 years old) and were likely not aware of the reasons (i.e., the memory of *Zamnè* as a famine food and historical heritage) behind the promotion of *Zamnè* as a traditional and cultural food [12,27]. In fact, the knowledge of traditional foods such as *Zamnè* is rapidly disappearing, and it is essential to reinforce the education of the young generation, safeguard those cultural heritages, and underpin food diversity, which is essential to support healthy diets and maintain environmental stewardship and sustainability [12,28,29].

Nevertheless, as shown in Section 3.2, the sensory appeal of *Zamnè* and *pseudoZamnè* should have been key to their maintenance in human diets, compared to many related species, which remain solely famine/emergency foods [2,3,30]. Actually, the off-taste (bitterness, sourness, and aftertaste) of some *Senegalia* seed species (e.g., *Zamnè* and *S. erythrocalyx*) (Section 3.4) should first be reduced, masked, or eliminated before their promotion as foods. The a priori perception that the traditionally cooked *Zamnè* is bitter (Table 2) could be explained by the eventual mix-up of *Zamnè* with other seed species or *pseudoZamnès* [9,27], indicating the need for more control of fraud in the market. It is important to highlight that it was not possible to find a report on human consumption of *S. erythrocalyx* seeds. The persistence of their bitterness indicates that the traditional cooking was not sufficient to eliminate the potentially toxic components (i.e., hemolytic compounds) in *S. erythrocalyx* seeds [6]. As shown, alternative processing to the traditional cooking procedure will be necessary to make *S. erythrocalyx* seeds palatable and edible for humans. *S. erythrocalyx* was included in this study because of its conventional cooking quality and particular compositional properties (e.g., saponins and starch content) compared to *Zamnè* and *pseudoZamnè* [31].

*Zamnè* and *pseudoZamnè* are mainly merely boiled and served with only oil and salt in Burkina Faso, and they were tested as such in the present study. However, considering the subtle taste of the traditionally cooked *Zamnè* and the *pseudoZamnè*, they could be used in diverse food preparations to add some unique flavors. For instance, the *pseudoZamnè* is more often prepared as a stew (known as *Panchkuta*) in India [1,4], and *Zamnè* is being prepared more and more similarly as well [24], which could improve their overall sensory appeal. Moreover, the green *Zamnè* is mainly nibbled during foraging or used to prepare a traditional sauce (i.e., Kari) in the Yadcé ethnic group in Burkina Faso. The unfamiliarity of the study participants with the (blanched) green *Zamnè* could explain why they disliked it. It will be essential to develop and disseminate alternative processing methods for *Senegalia* seeds in order to promote them in human diets.

Considering the hard-to-cook problem of *Senegalia* seeds (which implies a long cooking process and products of low nutritional quality) [9,10], *Zamnè* (as a prototype) was processed into tempeh in our previous study [5]. As an outcome, the fermentation into tempeh improved its nutritional properties and digestibility [5]. Subsequently, the present study aimed to determine the sensory appeal of the newly developed tempeh products from *Zamnè*. As shown, there is a considerable opportunity to promote *Zamnè* or *Senegalia* seeds’ tempeh if their nutritional properties and culinary uses are well disseminated (Section 3.1). However, tempeh is not yet well known in West Africa (including Burkina Faso) [11,32], justifying why it was only moderately appreciated in the present study (Section 3.2). It is worth noting that the earliest evaluation of the acceptance of tempeh in West Africa was 20 years ago, and the food culture might have changed since then. But still, the fact remains that tempeh could be processed into functional food products (e.g., stew, infant flour, beverage, and bakery products) that can hide its original appearance and improve its acceptability [11,33]. Therefore, attention should be paid to the drying or dehydration of *Zamnè* tempeh, which was associated with the development of a soumbala-like taste and a persistent aftertaste (Table S2). Further investigation, with a larger number of participants, is needed to determine the factors (e.g., sociodemographic profiles, food cultures, and neophobia scales) behind the variations in the appreciation of tempeh products [11,12].

Last but not least, to the best of our knowledge, this study is the first to provide data on the sensory properties and appeal of *Senegalia* seed foods. Also, there are a limited number of reports on the sensory properties of related *Acacia* s.l. seed foods. Only a few studies describe the sensory properties of roasted *Acacia* s.l. seeds and their blended flours [26,34,35,36], limiting any sensory matching with all of the products presented in the present study. Only the developed fresh *Zamnè* tempeh had a relatively good sensory match (especially the subtle nutty flavor) to fresh soy tempeh [12,37]. Suffice it to say that *Zamnè* could be a promising substrate for tempeh production. However, it is important to clarify that, as a consumer-based study, a list of general sensory descriptors was provided to our (untrained) participants, and most of them resolved themselves by describing all of the products as unique in taste (Table S2), likely due to their difficulty in associating them with other products that they knew, as well as their limited understanding of the sensory vocabulary. A further study, particularly with trained sensory assessors, is needed to refine the lexicon and determine the detailed sensory profiles of the products.

## 5. Conclusions

*Senegalia* seeds are wild and promising healthy legumes in the arid and semi-arid tropics. The present study provides unprecedented insight into their sensory and culinary properties. The seed species *Zamnè* and *Kumatiya* or *pseudoZamnè* demonstrated unique sensory attributes that could facilitate their promotion in human diets. In contrast, the related seed species *S. erythrocalyx*, traditionally cooked and explored for its food potential, proved to be unpalatable (and likely inedible), illustrating the variability in the culinary and sensory properties of *Senegalia* seeds. Overall, the present study provides essential data that will facilitate the valorization and branding of *Senegalia* seeds. It will be essential to disseminate the food uses of *Senegalia* seeds and develop alternative processing methods that align with the food culture and the sensory preferences of the targeted consumers.

## Figures and Tables

**Table 1:** Panel composition.

Information on the Participants		N	%
Total participants		80	100
Gender			
	Female	49	61
	Male	31	39
Age (years)			
	19–30	55	69
	31–40	21	26
	41–53	4	5
Fans of *Zamnè*		31	39
Frequency of consumption of *Zamnè*			
	At least once every week	3	4
	At least once every month	15	19
	At least once every year	25	31
	Very occasional	35	43
	Tried only once	2	3
Any knowledge of tempeh before this study?			
	Yes	2	3
	No	78	97

% = percentage of the total participants.

**Table 2:** A priori attitudes to the traditionally cooked *Zamnè* and expectations from any alternative processing.

Descriptors		n	%
Reasons for liking the traditionally cooked *Zamnè* (N = 31)			
	Taste	14	45
	Nutritional properties	11	35
	Culture	8	26
	Wholesomeness	4	13
	Medicinal properties	2	6
	Not responded	5	16
Reasons for disliking the traditionally cooked *Zamnè* (N = 49)			
	Low accessibility	16	33
	Tasteless	11	22
	Bitterness	8	16
	Smell	2	4
	Appearance	1	2
	Lack of nutritional information	1	2
	Cooking labor	1	2
	Not responded	11	22
Expectations from any novel product from *Zamnè* (N = 80)			
	Higher nutritional properties	30	38
	Health benefits	23	29
	Easy to prepare	19	24
	Affordability	2	3

The table summarizes the responses to a check-all-that apply questionnaire and open-ended comments; n = number of respondents and % = percentage of the total participants (N).

## Data Availability

The data generated for this study are available upon request to the corresponding author.

## Supplementary Materials

The following supporting information can be downloaded at: https://www.mdpi.com/article/10.3390/foods12234268/s1, Table S1: Sensory appeal and overall perceptions (mean scores ± standard deviation) of the products; Table S2: Frequencies (%) of use of the sensory descriptors by the participants (N = 77); Table S3: Differences between the clusters in the frequencies (%) of use of the sensory descriptors; Table S4: Interrelationships between the sensory attributes and the overall appeal of the products ^Ω^.
