# Supplementary material for: Non-Sensory Perception and Sensory Appeal of Zamnè, PseudoZamnè, Traditionally Cooked Senegalia erythrocalyx Seeds, and Tempeh According to Burkinabe Consumers
Source: Foods. 2023 Nov 26;12(23):4268. doi: 10.3390/foods12234268 (PMC10706274; doi:10.3390/foods12234268)
Supplement: Supplementary file 1 [file foods-12-04268-s001.zip › foods-2689977-supplementary.pdf]

foods

MDPI

Supplementary Materials

Article

Non-sensory perception and sensory appeal of *Zamnè*, *pseudoZamnè*, traditionally cooked *Senegalia erythrocalyx* seeds, and tempeh According to Burkinabe consumers

Moustapha Soungalo Drabo [1,3], Korotimi Traore [2], Charles Parkouda [2], Fatoumata Hama-Ba [2], Aly Savadogo [3] and Katleen Raes [1*]

Table S1. Sensory appeals and overall perceptions (mean scores ± standard deviation) of the products

	Products							
Attribute	TCZ	CSs	CSe	RZ	fST	fZT	dZT	p-value^
Sensory appeal								
Aroma	6.8 ±1.9^{a}	6.2 ±2.1^{ab}	5.4 ±2.0^{bc}	4.4 ±2.3^{c}	4.5 ±2.1^{c}	5.0 ±2.3^{c}	5.3 ±2.5^{bc}	<0.001
Appearance	6.5 ±1.8^{a}	7.0 ±1.9^{a}	5.2 ±2.1^{b}	5 ±2.1^{bc}	4.8 ±2.2^{bc}	4.7 ±2.1^{bc}	4.1 ±2.1^{c}	<0.001
Texture/mouthfeel	6.7 ±1.7^{a}	6.9 ±1.7^{a}	4.9 ±2.1^{bc}	4.8 ±2.1^{bc}	5.4 ±2.1^{c}	4.9 ±2.1^{bc}	4.2 ±2.1^{b}	<0.001
Taste	6.4 ±1.8^{a}	6.0 ±2.0^{ab}	3.4 ±2.3^{c}	3.4 ±1.8^{c}	5.0 ±2.3^{bd}	4.9 ±2.2^{d}	4.6 ±2.1^{d}	<0.001
Aftertaste	6.2 ±2.0^{a}	6.0 ±2.2^{a}	3.1 ±2.1^{b}	3.8 ±2.2^{bc}	5.5 ±2.2^{ad}	5.3 ±2.0^{ad}	4.7 ±2.2^{cd}	<0.001
Overall appeal (whole panel, N = 77)	7.0 ±1.5^{a}	6.5 ±1.8^{a}	4.0 ±2.3^{bc}	3.8 ±2.0^{b}	5.2 ±2.0^{d}	4.9 ±2.0^{cd}	4.8 ±2.3^{cd}	<0.001
Overall appeal (cluster 1, n = 40)	6.6 ±1.4a*	6.4 ±1.7$^{a\Omega}$	3.3 ±1.9$^{b\Omega}$	3.6 ±1.6$^{b\Omega}$	5.8 ±1.5$^{ac\Omega}$	5.3 ±1.4$^{c\Omega}$	5.9 ±1.6^{ac*}	<0.001
Overall appeal (cluster 2, n = 25)	7.2 ±1.7^{a*}	6.1 ±2.0$^{a\Omega}$	4.1 ±2.4$^{bc\Omega}$	2.7 ±1.3$^{b\Omega}$	3.4 ±1.6$^{d\Psi}$	3.1 ±1.7$^{cd\Psi}$	2.5 ±1.4$^{cd\Omega}$	<0.001
Overall appeal (cluster 3, n = 12)	7.7 ±0.9a*	8.1 ±1.0^{a*}	6.3 ±2.1^{ab*}	7.0 ±1.0^{ab*}	7.2 ±1.2^{ab*}	7.3 ±1.0^{ab*}	5.6 ±2.7^{b*}	0.009
p-value^^	0.037	0.008	0.003	<0.001	<0.001	<0.001	<0.001	<0.001$^{\Sigma}$
Overall perceptions								
Willingness to buy	4.1 ±1.0^{a}	4.0 ±1.1^{a}	2.27 ±1.37^{bc}	2.2 ±1.3^{b}	3.0 ±1.3^{d}	2.8 ±1.2^{bcd}	2.8 ±1.5^{cd}	<0.001
Willingness to eat if "last resort"	4.6 ±0.6^{a}	4.4 ±0.8^{a}	2.94 ±1.46^{b}	2.9 ±1.4^{b}	3.58 ±1.2^{c}	3.6 ±1.2^{c}	3.6 ±1.3^{c}	<0.001

TCZ, CSs, and CSe refer to the traditionally cooked *Zamnè* (*Senegalia macrostachya* seeds), *pseudoZamnè* (*Senegalia senegal* seeds), and *Senegalia erythrocalyx* seeds, respectively. RZ, fST, fZT, and dZT refer to the blanched green *Zamnè*, fresh soy tempeh, fresh *Zamnè* tempeh, and *Zamnè* tempeh crisps, respectively. The clusters were identified by performing K-mean clustering based on the scoring of the aroma, appearance, texture, taste, aftertaste, and overall sensory appeals of the products. The sensory appeals were scored between 1 = “dislike extremely” and 9 = “like extremely”, and the overall perceptions were scored between 1 = “certainly will not buy/eat even if last resort” and 5 = “certainly will buy/eat if last resort”. The p-values were derived from ANOVA (with $^{\Sigma}$the product and the cluster as interacting factors, and the ^product and the ^^cluster as independent factors). Scores in the same row and with different letter (a-d) superscripts were considered significantly different at $p < 0.05$ (Tukey range test). The scores of the overall appeal in the same column and with different symbols ($^{*, \Omega, \Psi}$) were considered significantly different at $p < 0.05$ (Tukey range test).

foods MDPI

Table S2. Frequencies (%) of use of the sensory descriptors by the participants (N = 77)

	Products							
Descriptors	TCZ	CSs	CSe	RZ	fST	fZT	dZT	p-value^
Unique taste	34^{a}	32^{a}	53^{ab}	59^{b}	47^{ab}	43^{ab}	36^{a}	<0.001
Tasteless	53^{a}	54^{a}	14^{b}	11^{b}	42^{a}	45^{a}	36^{ab}	<0.001
Nutty	9^{a}	11^{a}	8^{a}	11^{a}	9^{a}	32^{b}	8a	<0.001
Soumbala-like taste	0^{a}	3^{a}	5^{a}	0^{a}	3^{a}	3^{a}	39^{b}	<0.001
Fish-like taste	0a	5^{a}	1^{a}	5^{a}	11^{a}	9^{a}	12^{b}	0.004
Potash/alkaline taste	24^{a}	13^{ab}	28^{a}	4^{b}	9^{b}	5^{b}	12^{ab}	<0.001
Soapy taste	5^{a}	12^{a}	12^{a}	4^{a}	20^{a}	18^{a}	12^{a}	0.019
Sour	0^{a}	3^{a}	5^{a}	51^{b}	8^{a}	4^{a}	8^{a}	<0.001
Bitter	0^{a}	1^{a}	26^{b}	16^{ab}	5^{a}	4^{a}	9^{ab}	<0.001
Astringent	3	1	11	9	5	4	3	0.073
Persisting after taste	11^{a}	9^{a}	33^{b}	18^{ab}	14^{ab}	17^{ab}	25^{ab}	0.003
Dull appearance	63^{a}	30^{b}	70^{a}	18^{b}	58^{a}	28^{b}	72^{a}	<0.001
Appealing appearance	32^{ab}	59^{c}	17^{a}	67^{c}	30^{ab}	54^{bc}	13^{a}	<0.001
Soumbala appearance	50^{a}	49^{ad}	28^{bc}	11^{bc}	28^{bc}	4^{b}	30^{cd}	<0.001
Soft	62^{a}	54^{ab}	37^{ab}	22^{b}	57^{a}	46^{ab}	7^{c}	<0.001
Greasy	9^{a}	12^{a}	34^{b}	9^{a}	11^{a}	13^{a}	0^{a}	<0.001
Hard	4^{a}	11^{ab}	4^{a}	21^{bc}	4^{a}	5^{a}	47^{c}	<0.001
Dry	3^{a}	0^{a}	0^{a}	5^{a}	3^{a}	1^{a}	53^{b}	<0.001
Crispy	11^{ab}	7^{a}	5^{a}	12^{ab}	4^{a}	5^{a}	25^{b}	<0.001
Grainy/earthy	8^{ab}	8^{ab}	14^{ab}	18^{a}	14^{ab}	13^{ab}	0^{b}	0.006

TCZ, CSs, and CSe refer to the traditionally cooked *Zamnè* (*Senegalia macrostachya* seeds), *pseudoZamnè* (*Senegalia senegal* seeds), and *Senegalia erythrocalyx* seeds, respectively. RZ, fST, fZT, and dZT refer to the blanched green *Zamnè*, fresh soy tempeh, fresh *Zamnè* tempeh, and *Zamnè* tempeh crisps, respectively. ^The p-values were derived from Cochran-Q test with the product as the factor. The frequencies in the same row but with different letter (a-d) superscripts were considered significantly different based on Bonferroni-corrected p-values (Dunn test).

Table S3. Differences between the clusters in the frequencies (%) of use of the sensory descriptors

	TCZ			CSs			CSe			RZ		
Descriptors	C2~	C3~	p-value^	C2~	C3~	p value^	C2~	C3~	p-value^	C2~	C3~	p-value^
Unique taste	34	28	0.406	37	24	0.215	61	40	0.080	61	48	0.219
Tasteless	41	64	0.064	**44**	**72**	**0.024**	15	20	0.404	10	16	0.351
Nutty	10	12	0.538	17	12	0.428	5	4	0.681	7	16	0.239
Soumbala-like taste	0	0		2	4	0.14	5	4	0.681	0	0	
Fish-like taste	0	0		2	4	0.14	0	4	0.379	2	8	0.319
Potash/alkaline taste	**37**	**12**	**0.026**	20	8	0.404	**15**	**36**	**0.045**	2	4	0.14
Soapy taste	2	4	0.14	15	4	0.173	17	8	0.256	5	4	0.681
Sour	0	0		2	4	0.14	5	4	0.681	54	44	0.306
Bitter	0	0		2	0	0.621	34	20	0.171	15	16	0.572
Astringent	0	4	0.379	0	4	0.379	12	8	0.462	7	8	0.633
Persisting after taste	10	12	0.538	7	4	0.510	29	32	0.513	20	16	0.495
Dull appearance	66	48	0.121	34	28	0.406	68	64	0.461	17	20	0.505
Appealing appearance	24	44	0.094	56	60	0.170	10	24	0.114	63	64	0.234
Soumbala appearance	46	52	0.424	39	60	0.080	17	32	0.136	7	16	0.239
Soft	66	56	0.294	54	44	0.306	41	28	0.201	20	20	0.648
Greasy	10	8	0.59	12	16	0.464	29	44	0.170	12	4	0.256
Hard	2	8	0.319	10	16	0.351	2	4	0.14	22	28	0.393
Dry	2	0	0.621	0	0		0	0		2	8	0.319
Crispy	2	16	0.064	2	8	0.319	2	12	0.148	10	8	0.59
Grainy/earthy	10	4	0.367	10	4	0.367	10	12	0.538	17	24	0.352

	fST			fZT			dZT		
Descriptors	C2~	C3~	p-value^	C2~	C3~	p value^	C2~	C3~	p-value^
Unique taste	46	52	0.4236	41	36	0.4301	29	40	0.2638
Tasteless	**34**	**60**	**0.0361**	39	48	0.323	27	36	0.3029
Nutty	10	8	0.5901	34	28	0.4055	10	0	0.1405
Soumbala-like taste	5	0	0.3823	5	0	0.3823	46	28	0.1107

Fish-like taste	10	12	0.5381	7	12	0.4307	**5**	**28**	**0.0119**
Potash/alkaline taste	5	8	0.4897	5	8	0.4897	12	16	0.4644
Soapy taste	27	8	0.05687	17	20	0.5048	7	24	0.06291
Sour	5	8	0.4897	2	4	0.14	2	12	0.1484
Bitter	5	8	0.4897	2	0	0.6212	15	4	0.1732
Astringent	10	0	0.1405	0	12	0.05026	2	0	0.6212
Persisting after taste	17	16	0.5963	17	20	0.5048	29	20	0.2963
Dull appearance	56	48	0.3499	27	20	0.3747	66	76	0.2787
Appealing appearance	29	36	0.3806	54	56	0.5284	15	12	0.5356
Soumbala appearance	22	24	0.4236	5	4	0.6809	34	24	0.2787
Soft	63	52	0.2552	44	40	0.4796	5	4	0.6809
Greasy	5	20	0.06588	15	16	0.5722	0	0	
Hard	0	4	0.379	2	4	0.1401	51	40	0.2643
Dry	2	0	0.6212	0	0		51	56	0.4515
Crispy	2	8	0.319	5	4	0.6809	**100**	**24**	**<0.001**
Grainy/earthy	12	16	0.4644	15	12	0.5356	0	0	

Table S4. Inter-relationships between the sensory attributes and the overall appeal of the products[Ω]

	Appearance	Texture	Taste	Aftertaste	Over. appeal	Wtb	Wte
Aroma	0.77*	0.75	0.74	0.55	0.82*	0.84*	0.83*
Appearance		0.96***	0.65	0.52	0.77*	0.79*	0.73
Texture			0.80*	0.70	0.88*	0.88*	0.84*
Taste				0.96***	0.98***	0.97***	0.98***
Aftertaste					0.91**	0.89*	0.91**

[Ω] Seventy-seven (77) participants scored the products, and the Pearson correlation coefficients between the scores were determined. Over. appeal, Wtb, Wte, and the texture refer to the overall appeal, the willingness to buy, the willingness to eat if the products were the last resort (*i.e.*, during a famine), and the mouthfeel, respectively. The correlation coefficients (r) with *, **, and *** had p-values below 0.05, 0.01, and 0.001, respectively.
